# Supplementary material for: Engineered Peptides Enable Biomimetic Route for Collagen Intrafibrillar Mineralization
Source: Int J Mol Sci. 2023 Mar 28;24(7):6355. doi: 10.3390/ijms24076355 (PMC10093982; doi:10.3390/ijms24076355)
Supplement: Supplementary file 1 [file ijms-24-06355-s001.zip › ijms-2162306-supplementary.pdf]

Engineered Peptides Enable Biomimetic Route for Collagen Intrafibrillar Mineralization

Aya Cloyd [1,2], Kyle Boone [2,3], Qiang Ye [2], Malcolm L. Snead [4], Paulette Spencer [1,2,3] and Candan Tamerler [1,2,3,*]

[1] Bioengineering Program, University of Kansas, Lawrence, KS 66045, USA
2 Institute for Bioengineering Research, University of Kansas, Lawrence, KS 66045, USA
3 Department of Mechanical Engineering, University of Kansas, Lawrence, KS 66045, USA
4 Center for Craniofacial Molecular Biology, Herman Ostrow School of Dentistry of USC, University of Southern California, Los Angeles, CA 90007, USA

* Correspondence: ctamerler@ku.edu

Supplemental Table S1. Biochemical properties of peptides
Calculated using ExPasy ProtParam tool [47]

Peptide name & chemical properties	Peptide Sequence
**CBP**<br>MW: 960.19 g/mol<br>Theoretical pI: 11.17<br>Gravy Index: -0.850	TKKLTLRT
**HABP1**<br>MW: 761.90 g/mol<br>Theoretical pI: 6.69<br>Gravy Index: -0.129	MLPHHGA
**CBP-(linker)-HABP1 (CBP-HABP1)**<br>MW: 1943.34<br>Theoretical pI: 11.17<br>Gravy Index: -0.32	TKKLTLRT-(APA)-MLPHHGA

Supplemental Table S2. Statistics on PeakForce-QNM DMT Modulus pre/post mineralization

	Pre-mineralization			
	**Collagen**	**Collagen-(CBP)**	**Collagen-(HABP1)**	**Collagen-(CBP-HABP1)**
Mean (GPa)	4.08	5.16	4.62	5.25
Standard Deviation (GPa)	0.010	0.018	0.057	0.039
Variance (GPa^2)	0.065	0.215	2.16	1.01
	**Post-mineralization**			
	**Collagen**	**Collagen-(CBP)**	**Collagen-(HABP1)**	**Collagen-(CBP-HABP1)**
Mean (GPa)	6.96	6.52	6.73	5.89
Standard Deviation (GPa)	0.483	0.107	1.708	0.140
Variance (GPa^2)	2.34	11.4	2.92 x 10^3	19.6

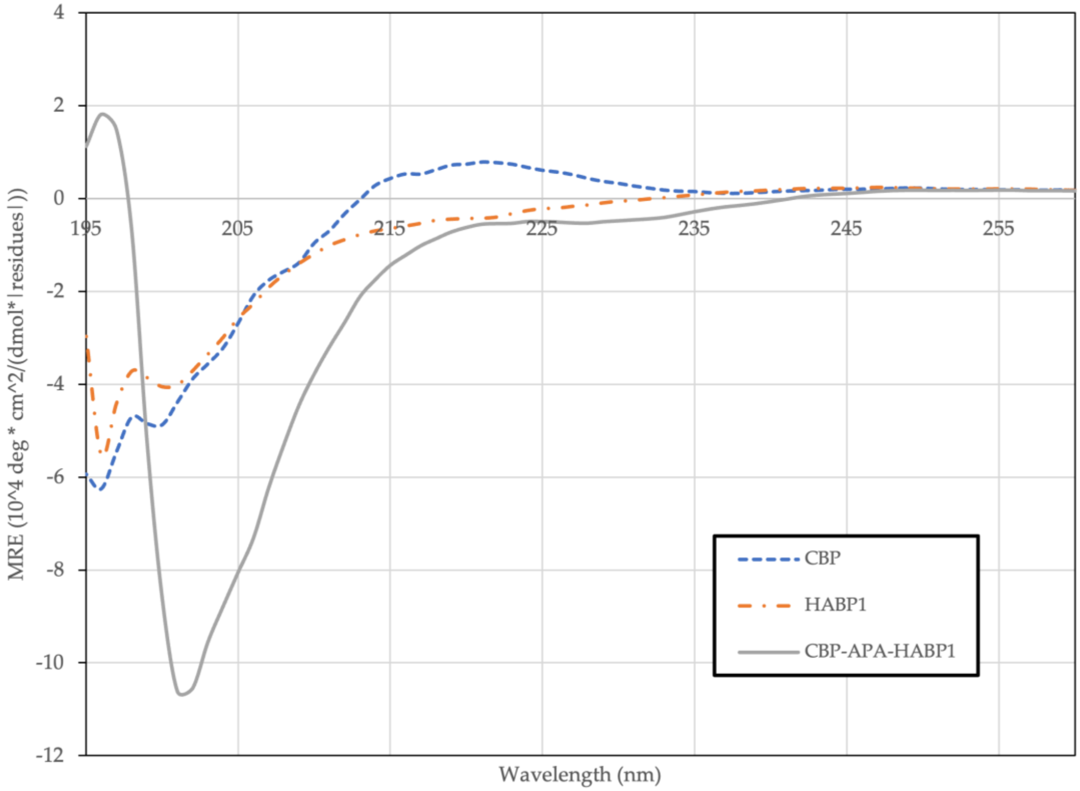

Supplemental Figure S1. Mean residue ellipticity (MRE) by circular dichroism of collagen binding peptide (CBP), hydroxyapatite peptide (HABP1) and bifunctional peptide (CBP--APA-HABP1), computed using CD Pro [52].

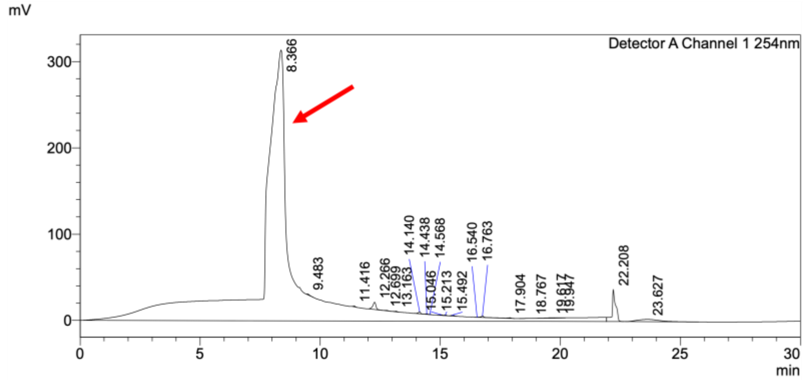

Detector A Channel 1 254nm

Peak#	Ret. Time	Area	Height	Area%
1	8.366	28688449	313746	97.430
2	9.483	6459	1163	0.022
3	11.416	5953	1157	0.020
4	12.266	76256	8430	0.259
5	12.699	2018	301	0.007
6	13.163	5126	663	0.017
7	14.140	16273	2596	0.055
8	14.438	4835	721	0.016
9	14.568	1907	399	0.006
10	15.046	4704	698	0.016
11	15.213	4887	827	0.017
12	15.492	10072	998	0.034
13	16.540	1490	221	0.005
14	16.763	12733	1998	0.043
15	17.904	11223	709	0.038
16	18.767	2219	104	0.008
17	19.617	11966	390	0.041
18	19.947	8693	420	0.030
19	22.208	392014	37134	1.331
20	23.627	178050	2676	0.605
Total		29445327	375350	100.000

Supplemental Figure S2. Collagen binding peptide (CBP) analytical Shimadzu HPLC spectral report, red arrow signifying CBP spectral feature.

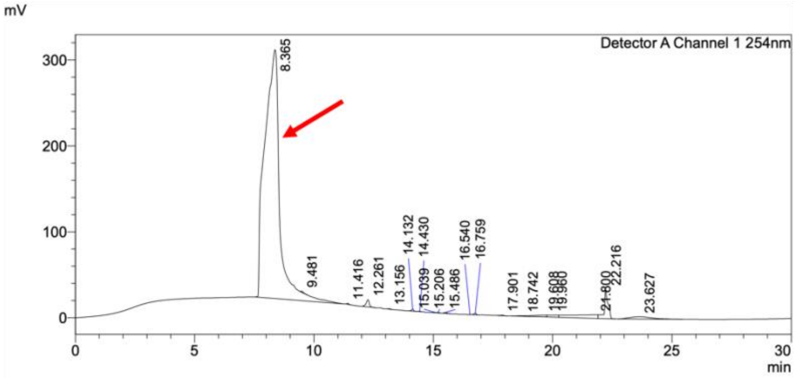

Detector A Channel 1 254nm

Peak#	Ret. Time	Area	Height	Area%
1	8.365	12515209	289474	91.360
2	9.481	7493	1410	0.055
3	11.416	5815	1146	0.042
4	12.261	74582	8327	0.544
5	13.156	3367	620	0.025
6	14.132	14721	2557	0.107
7	14.430	2943	566	0.021
8	15.039	2816	539	0.021
9	15.206	2864	629	0.021
10	15.486	8300	909	0.061
11	16.540	1104	178	0.008
12	16.759	11785	1961	0.086
13	17.901	4707	674	0.034
14	18.742	12545	668	0.092
15	19.608	79143	1965	0.578
16	19.960	71676	2409	0.523
17	21.800	328776	4232	2.400
18	22.216	375565	34413	2.742
19	23.627	175361	2678	1.280
Total		13698776	355356	100.000

Supplemental Figure S3. Hydroxyapatite binding peptide (HABP1) analytical Shimadzu HPLC spectral report, red arrow signifying HABP1 spectral feature.

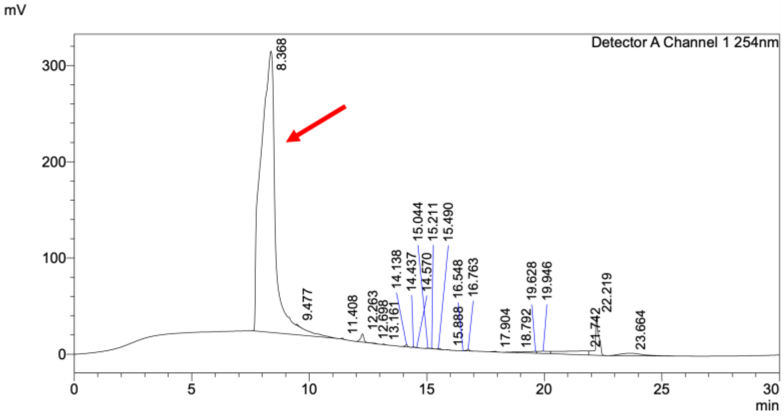

Detector A Channel 1 254nm

Peak#	Ret. Time	Area	Height	Area%
1	8.368	12624281	292235	91.182
2	9.477	6024	1261	0.044
3	11.408	5467	1091	0.039
4	12.263	77440	8536	0.559
5	12.698	3537	229	0.026
6	13.161	3131	602	0.023
7	14.138	14991	2548	0.108
8	14.437	2624	525	0.019
9	14.570	1160	296	0.008
10	15.044	2843	548	0.021
11	15.211	2963	636	0.021
12	15.490	8286	917	0.060
13	15.888	1032	192	0.007
14	16.548	1395	211	0.010
15	16.763	12233	1963	0.088
16	17.904	4459	645	0.032
17	18.792	13848	707	0.100
18	19.628	67905	1985	0.490
19	19.946	82721	2385	0.597
20	21.742	322265	4146	2.328
21	22.219	416607	40621	3.009
22	23.664	169928	2603	1.227
Total		13845142	364879	100.000

Supplemental Figure S4. Chimeric peptide (CBP-HABP1) analytical Shimadzu HPLC spectral report, red arrow signifying CBP-HABP1 spectral feature.

```python
import numpy as np
from glob import glob
from pathlib import Path
from PIL import Image
import os
import cv2
from tqdm import tqdm
from matplotlib import pyplot as plt
from skimage.filters import threshold_multiotsu



# Path to the data directory
main_dir = Path("/INSERT PATH HERE")

# Set all tif files in within the data dir..
tifs = []
for child in os.listdir(main_dir):
    print(child)
    tifs.append(list((main_dir / child).glob("*.tif")))


def area_precentage(image):
    '''Calculate the area of the pixels above a certain threshold is fixed to zero'''
    pixels = len(np.column_stack(np.where(image > 0)))
    image_area = image.shape[0] * image.shape[1]
    area_ratio = round((pixels / image_area) * 100,2)
    return area_ratio

def findArea (image, name):
    #apply blur to remove the small bright spots
    image = cv2.medianBlue(image, 7)

    #equalize histogram
    eq_image = cv2.equalizeHist(image.ravel()).reshape((image.shape[0],image.shape[1]))

    # Find the thresholds in the histogram
    # Set any value less than the smallest threshold to zero results in a binary image
    (threshold, regions) = cv2.threshold(image, 0, 1, cv2.THRESH_BINARY | cv2.THRESH_OTSU)[1].astype(np.bool)

    #calculate the area of the binary image
    area = area_precentage(regions)

    # Visualize results
    fig, ax = plt.subplots(nrows=1, ncols=3, figsize=(15, 3))
    im_ratio = regions.shape[0]/regions.shape[1]

    # Plotting the original image
    im1  = ax[0].imshow(image, cmap='gray')
    ax[0].set_title('Original')
    ax[0].axis('off')

    # Plotting the histogram and the corresponding thresholds
    im2 = ax[1].hist(eq_image.ravel(), bins=255)
    ax[1].set_title('Histogram')
    ax[1].axvline(threshold, color='r')

    # Plotting the binary image
    im3 = ax[2].imshow(regions, cmap='gray')
    ax[2].set_title('Binary image area {:.3f}'.format(area))
    ax[2].axis('off')

    fig.colorbar(im3, ax=ax[2], orientation="vertical", fraction=0.047*im_ratio)

    plt.tight_layout()
    plt.show()
    fig.savefig('INSERT PATH HERE' + name + '_{}.png'.format(area))
    return np.uint8(regions)

# Iterate through loaded images
for i in tqdm(tifs):
    for file in i:
        name = str(file).split('\\')[-1].split('.')[0]
        image = np.array(Image.open(file))[:1790,:] # PIL is the easiest library to load tif into python
        regions = findArea(image, name)
```

Supplemental Figure S5. SEM image processing source code (Python 3.9.13)
